# Impact of antigen identification on transplant free survival in interstitial lung disease

**DOI:** 10.1186/s12890-023-02724-w

**Published:** 2023-10-26

**Authors:** Margaret Kypreos, Kiran Batra, Craig S. Glazer, Traci N. Adams

**Affiliations:** 1 Division of Pulmonary and Critical Care Medicine, University of Texas Southwestern Medical Center, 5323 Harry Hines Boulevard, Dallas, TX 75219 USA; 2 Department of Radiology, University of Texas Southwestern Medical Center, Dallas, TX USA

**Keywords:** Hypersensitivity pneumonitis, Transplant free survival, Antigen identification, Interstitial lung Disease

## Abstract

**Introduction:**

Antigen identification impacts diagnosis as well as prognosis in patients with hypersensitivity pneumonitis. An antigen may also be present in other etiologies of interstitial lung disease, however it is unknown whether identification impacts survival.

**Methods:**

We evaluated a retrospective cohort in order to determine if antigen identification affects transplant free survival in patients with hypersensitivity pneumonitis, idiopathic pulmonary fibrosis, connective tissue disease interstitial lung disease, and interstitial pneumonia with autoimmune features. Only patients with definite or high probability of hypersensitivity pneumonitis by American Thoracic Society guidelines were included in the analysis.

**Results:**

Transplant free survival was improved with antigen identification in patients with hypersensitivity pneumonitis but not in patients with idiopathic pulmonary fibrosis, connective tissue disease interstitial lung disease, and interstitial pneumonia with autoimmune features.

**Conclusion:**

Our study suggests that removal of identified antigen in interstitial lung diseases other than hypersensitivity pneumonitis may not be impactful. Additionally, it further suggests that definitive diagnosis of hypersensitivity pneumonitis with bronchoalveolar lavage and transbronchial biopsy may be beneficial prior to recommending antigen removal.

**Supplementary Information:**

The online version contains supplementary material available at 10.1186/s12890-023-02724-w.

## Introduction

Interstitial lung diseases (ILD) are a group of diffuse parenchymal lung disease with various etiologies including environmental, occupational, drug related, idiopathic, and secondary to an underlying autoimmune disease. The overall mortality of interstitial lung disease is high with the highest rate of 2.5 per 100,000 people in the UK, Scandinavia, the Netherlands, and Spain. Over one hundred causes of ILD have been described, with the most common being underlying autoimmunity (as seen in connective tissue disease related ILD or interstitial pneumonia with autoimmune features) and fibrogenic exposures. Idiopathic ILD is also very common [1].

Hypersensitivity pneumonitis is a type of granulomatous ILD which involves repeated exposure to an inciting antigen. Identification of an inciting antigen improves transplant-free survival (TFS) in patients with HP, and removal of the inciting antigen is a cornerstone of treatment for those patients [2–4].

There is significant clinical, histopathologic, and mechanistic overlap between HP, CTD-ILD, and IPAF. Each of these conditions may have bronchocentric lymphocytic infiltrate histopathologically, though they can present with a variety of histopathologic patterns. Mechanistically, fibrotic HP, CTD-ILD, and IPAF produce activation of the same fibrotic cascade on a molecular level [5–9]. Finally, clinical data, including mortality data, show that populations of HP, CTD-ILD, and IPAF can be similar [7–10]. In fact, autoimmunity in patients with HP can portend a poor prognosis [11, 12]. Whether the reverse is true, that identification of antigen in patients with autoimmune-related ILD will impact survival, has not been previously evaluated. Due to the similarities between conditions, we hypothesize that identification of a fibrogenic exposure in ILDs other than HP, including CTD-ILD, IPF, and IPAF may impact survival. The objective of this retrospective study is to determine the impact of antigen identification on transplant free survival in patients with HP, IPF, CTD-ILD, and IPAF.

## Methods

We retrospectively identified ILD patients evaluated between 2003 and 2018 from the University of Texas Southwestern Medical Center (UTSW). This study was conducted in accordance with the amended Declaration of Helsinki, and the UTSW Institutional Review Board approved the study (STU-2019-0913). Patients who had a diagnosis of HP, CTD-ILD, IPAF, and IPF were included in this study. Patients were excluded if their ILD was not secondary to one of the above etiologies. The diagnosis of HP was determined by the American Thoracic Society guidelines and consensus from multidisciplinary discussion. Only patients with a definite or high probability of HP were included [13]. Providers use a detailed questionnaire to ascertain exposure (Supplementary Appendix 1). Avian antigen was documented if there was consistent exposure to a live bird or feather products. Mold antigen was documented if the patient was persistently exposed to visible mold either at home or in the office or consistently used compost heap. For all exposures, a temporal relationship was established, and exposures were only deemed significant if they were persistent and preceded the development of ILD. The exposure history was reviewed by an occupational medicine specialist in cases where it was unclear if the exposure was significant enough to lead to sensitization. Bronchoalveolar lavage (BAL) lymphocytosis was defined as greater than 30% lymphocytes. High resolution computed tomography (HRCT) images were reviewed by a thoracic radiologist who was blinded to the clinical diagnosis. The HRCT was defined as fibrotic and non fibrotic based on the presence or absence of reticulation and traction bronchiectasis.

Clinical data extracted from the medical record included age, gender, ethnicity, smoking history, potential fibrogenic antigen exposure, high resolution CT chest features, pulmonary function testing (PFTs), connective tissue disease serologies (CTD), completion of bronchoscopy, presence of BAL lymphocytosis, completion of transbronchial and surgical lung biopsy, gender-age physiology index, date of death, and date of transplant.

Continuous variables were expressed as means and standard deviations; comparisons were made using Student’s t test or Wilcoxon signed rank sum test as appropriate. Categorical variables were expressed using counts and percentages; comparisons were made using Chi-squared test or Fisher’s exact test, where appropriate. Survival analysis was done using Kaplan Meier curve. Univariable and multivariable Cox proportional hazard models were performed to evaluate transplant free survival in patients with and without antigen identification in patients with IPF, CTD-ILD, and IPAF. Variables in the univariable analysis have previously been associated with survival in ILD and include age, gender, FVC% predicted, DLCO% predicted, antigen exposure, smoking history, fibrosis on HRCT, need for supplemental oxygen, and GAP score. The variables that were significantly associated with change in diagnosis (p-value < 0.1) were included in the multivariable model to test independent associations. All p-values less than 0.05 were considered significant.

The primary outcome of this study was transplant free survival for patients with and without antigen identification in patients with IPF, CTD-ILD, IPAF, and HP.

## Results

### Patient characteristics

In our retrospective cohort of 724 patients with ILD, 110 (15.2%) met definite or high probability of HP, 86 (11.9%) were diagnosed with IPAF, 351 (48.5%) were diagnosed with CTD-ILD, and 177 (24.4%) were diagnosed with IPF. Demographic characteristics of the cohort are shown in Table 1. Mean age of the HP cohort was 64 years and 82% were Non Hispanic White. A potential fibrogenic exposure was identified in 80.9% of patients and 43% of patients underwent bronchoalveolar lavage. Transbronchial biopsy was completed in 46% of patients and surgical lung biopsy was completed in 53% of patients. Mean age of the CTD-ILD cohort was 54 years and 47% were Non Hispanic White. A potential fibrogenic exposure was identified in 21% of patients and 16% of patients underwent bronchoalveolar lavage. Transbronchial biopsy was completed in 10% of patients and surgical lung biopsy was completed in 21% of patients. Mean age of the IPAF cohort was 60 years and 64% were Non Hispanic White. A potential fibrogenic exposure was identified in 28% of patients and 20% underwent bronchoalveolar lavage. Transbronchial biopsy was completed in 13% of patients and surgical lung biopsy was completed in 35% of patients. Mean age of the IPF cohort was 68 years and 73% were Non Hispanic White. A potential fibrogenic exposure was identified in 28% of patients and 17% underwent bronchoalveolar lavage. Transbronchial biopsy was completed in 12% of patients and surgical lung biopsy was completed in 28% of patients.

### Transplant free survival

**Fig. 1:**
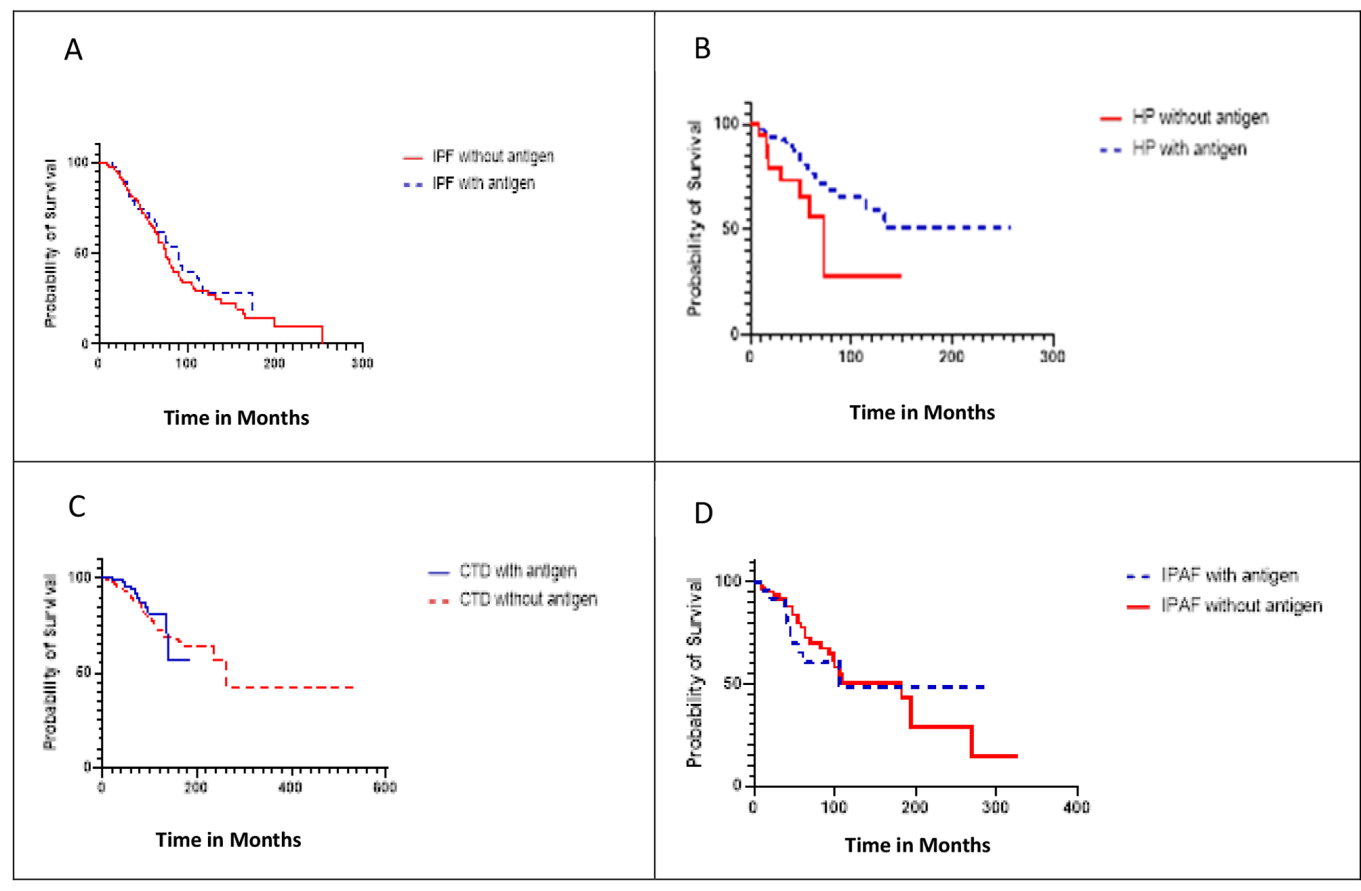
Kaplan Meier curves comparing transplant free survival in years in patients with IPF, HP, CTD-ILD, and IPAF with and without antigen identification. **A**) Survival of IPF with and without antigen; **B**) Survival of HP with and without antigen; **C**) Survival of CTD-ILD with and without antigen; **D**) Survival of IPAF with and without antigen

**Fig. 2:**
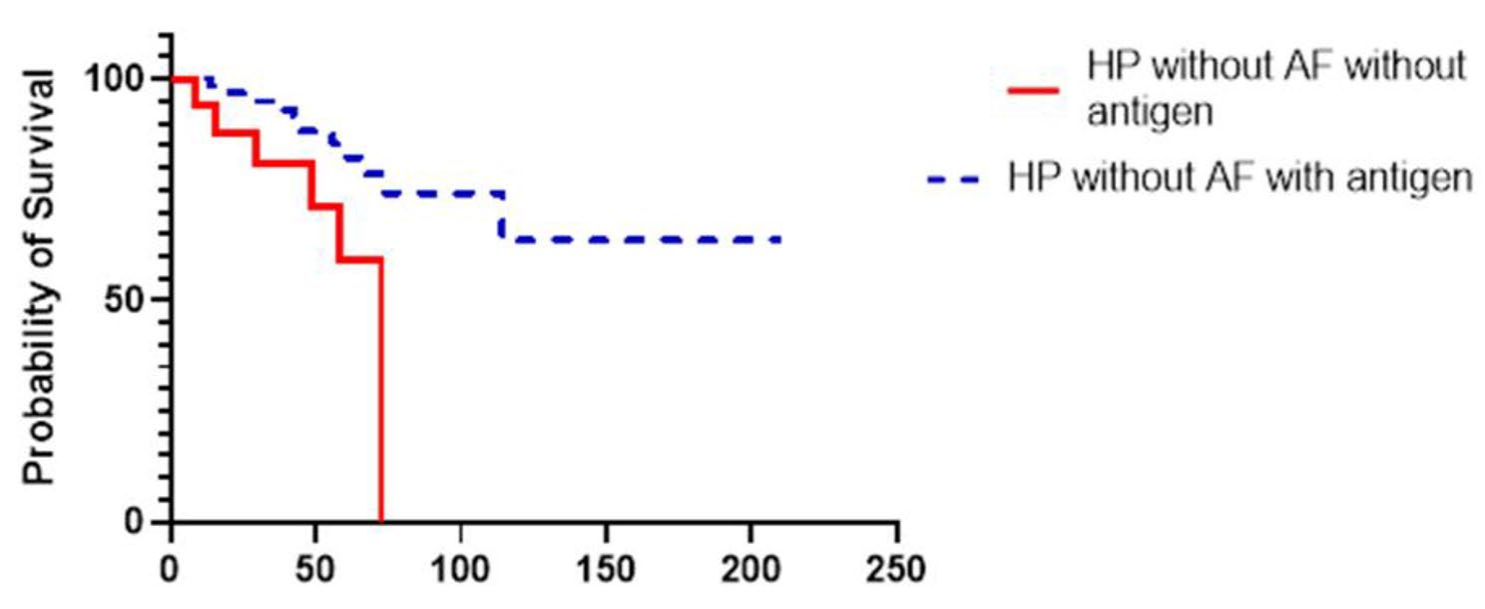
Kaplan Meier curve comparing transplant free survival in years in patients with HP without AF features with and without antigen identification

In a univariable model patients with definite or high probability HP, transplant free survival was found to be worse in those who also met criteria for IPAF (HR 2.06, 95% CI 0.95–4.33, p value = 0.06) (Table 2). Similar result was found in a multivariable model where survival was adjusted for smoking history, identified antigen, nonfibrotic HP, and the original GAP index (HR 2.97, 95% CI 1.34 to 6.50, p value = 0.007). GAP index was used in the multivariable analysis instead of age, FVC % predicted, DLCO % predicted, and gender due to insufficient events in our study to account for each variable individually. When identification of the antigen occurred in patients with IPF, there was no difference in transplant free survival (HR 1.14, 95% CI 0.74–1.74, p = 0.55) (Fig. 1). No difference in transplant free survival was identified among patients with CTD ILD (HR 0.89, 95% CI 0.49–1.58, p value = 0.69) as well as patients with IPAF (HR 1.12, 95% CI 0.52–2.39, p value = 0.77) (Fig. 1). This lack of difference in transplant free survival was also present when the CTD ILD patients were combined with the IPAF patients (HR 1.03, 95% CI 0.65–1.64, p value = 0.90). An improved transplant free survival with antigen identification was identified in the entire HP cohort in the multivariable analysis (HR 0.41, 95% CI 0.18–1.01, p = 0.04). In just the HP without IPAF cohort, antigen identification remained a significant predictor of TFS (HR 0.48, 95% CI 0.24–0.96, p = 0.02) (Fig. 2). We had insufficient patients with HP with IPAF to determine whether survival was impacted by presence of antigen.

**Table 1:** Demographic Characteristics of Retrospective Cohort

N = 724	HP N = 110	CTD N = 351	IPAF N = 86	IPF N = 177
Mean Age (SD)	64.1 (10.3)	54 (14.0)	60 (12.4)	68 (8.6)
Male, No. (%)	49 (44.5%)	68 (19.4%)	24 (27.9%)	130 (73.4%)
Ethnicity, No. (%)				
White	90 (81.8%)	165 (47.0%)	55 (64.0%)	132 (74.6%)
Black	5 (4.5%)	90 (25.6%)	11 (12.8%)	7 (4.0%)
Hispanic or Latino	7 (6.4%)	54 (15.4%)	10 (11.6%)	21 (11.9%)
Asian	6 (5.5%)	21 (6.0%)	3 (3.5%)	3 (1.7%)
Other	0 (0.0%)	2 (0.57%)	0 (0.0%)	0 (0.0%)
Unknown	2 (1.8%)	19 (5.4%)	7 (8.1%)	14 (7.9%)
Ever Smoker, No. (%)	41 (37.0%)	117 (33.3%)	34 (39.5%)	112 (63.3%)
Pack years, median (IQR)	20 (8.5–32.0)	12 (6.0–30.0)	18 (10.0–38.0)	20.5 (10–40)
Antigen Identified, No. (%)	89 (80.9%)	74 (21.1%)	24 (27.9%)	50 (28.2%)
Avian/Feather	57 (51.8%)	49 (14.0%)	14 (16.3%)	39 (22.0%)
Mold	46 (41.8%)	37 (10.5%)	12 (14.0%)	26 (14.7%)
Other **	10 (9.1%)	0 (0.0%)	2 (2.3%)	0 (0.0%)
Baseline Lung Function, mean				
FVC % predicted (SD)	67.0 (19.2)	68.4 (19.9)	64.5 (17.7)	74.4 (20.5)
DLCO% predicted (SD)	49.8 (17.5)	48.9 (20.2)	45.5 (16.7)	51.9 (22.6)
HRCT	110 (100%)	351 (100%)	86 (100%)	177 (100%)
Bronchoalveolar Lavage	43 (39.1%)	56 (15.9%)	17 (19.8%)	30 (16.9%)
Lung Biopsy Performed, No. (%)				
Transbronchial Biopsy	51 (46.3%)	35 (10.0%)	11 (12.8%)	22 (12.4%)
Surgical Lung Biopsy	58 (52.7%)	76 (21.7%)	30 (34.9%)	49 (27.7%)
Clinical Outcomes				
Death	8 (7.3%)	55 (15.7%)	27 (31.4%)	67 (37.9%)
Average Time to Death (Years)	3.64	6.32	5.44	6.05
Transplant	13 (11.8%)	20 (5.7%)	7 (8.1%)	36 (20.3%)
Average Time to Transplant (Years)	4.40	7.55	6.68	4.24

Other antigens include isocyanate exposure and fish tank exposure

**Table 2:** Univariable and multivariable analysis of TFS in HP using Cox proportional hazard models (N = 110)

Variable	Univariable analysis			Multivariable analysis		
	HR for death or transplant	95% CI	P value	HR for death or transplant	95% CI	P value
Age	0.979	0.94–1.02	0.30			
Never smoker	2.73	1.18–7.39	0.029	4.445	1.781 to 13.11	0.0029
Antigen	0.44	0.20–1.06	0.05	0.41	0.18–1.01	0.0394
FVC % predicted	0.96	0.93–0.98	< 0.0001			
DLCO % predicted	0.95	0.92–0.97	< 0.0001			
Absence of fibrosis	0.28	0.05–0.97	0.09	0.1542	0.02272 to 0.5883	0.0188
HP meeting IPAF criteria	2.06	0.95–4.33	0.06	2.974	1.324 to 6.499	0.0067
Oxygen use	1.74	0.78–3.73	0.16			
Female gender	0.57	0.27–1.24	0.15			
GAP index	1.37	1.04–1.82	0.03	1.214	0.9384 to 1.610	0.1571

## Discussion

In this study, we examined the impact of antigen identification on transplant free survival in patients with HP, IPF, CTD-ILD, and IPAF. Transplant free survival was found to be worse among patients with definite or high probability HP who met IPAF criteria and this finding persisted on multivariable analysis. With regard to identification of antigen, transplant free survival was improved in patients with HP and HP without autoimmune features. There was no difference in survival in patients with IPF, CTD-ILD, or IPAF. Our data does not suggest that antigen drives progression in IPF, CTD-ILD, and IPAF.

Our results suggest that it may be important to distinguish between HP, CTD ILD, and IPAF prior to recommending removal of antigen. Because the cornerstones of treatment of HP, CTD, and IPAF include immunosuppression and addition of nintedanib for progressive fibrotic disease, it remains controversial whether a tissue diagnosis is needed to distinguish between the diagnoses [3, 14–16]. Our results demonstrate that making a definitive diagnosis of HP may be helpful prior to recommending antigen removal, as antigen identification leads to improved TFS in patients with HP but not those with IPAF or CTD-ILD. This finding is presumed to be due to antigen removal in patients in whom antigen is identified, though causation between antigen removal and survival has not been definitively established [2, 16]. Petnak et al. demonstrated that both antigen identification and removal are associated with decreased all cause mortality and transplantation in fibrotic hypersensitivity pneumonitis [17]. Thus, determining a definitive diagnosis of HP may allow the clinician to more confidently determine that antigen identification and removal are indicated, especially when the latter is not easily accomplished by the patient [17, 19]. This determination can often be made with bronchoalveolar lavage and transbronchial biopsy, which carries minimal risk to the patient and has a yield in HP of up to 59% [13, 18]. Surgical lung biopsy carries a much higher risk and may not be warranted purely to determine the importance of antigen removal between HP and IPAF, but it is routinely used to distinguish HP from IPF, where treatment differences are profound [13, 18].

Our study demonstrated the same decreased survival with identification of autoimmune features in HP patients as Adegunsoye et al. The study demonstrated that the presence of autoimmunity in patients with chronic fibrotic HP may portend a poor prognosis, however the identification of the inciting antigen had on impact on survival. In that study, the diagnosis of HP was determined by multidisciplinary evaluation and tissue diagnosis was only obtained in 74 patients. Poorly formed granulomas were not required on histopathology and consistency with HP was considered if lymphocytic predominant interstitial infiltrate in a bronchiolocentric pattern was present [11]. Thus, it is unclear if the HP patients met definite or high probability criteria as confidence can be decreased to moderate if probable histology is present in association with an indeterminate HRCT. Additionally, it is unclear if their diagnosis was more consistent with CTD-ILD or IPAF with antigen identified. Further studies with HP patients diagnosed via the American Thoracic Society Guidelines are required in order to determine if survival in HP patients with autoimmune features is impacted by antigen identification [17].

Strengths of our study include the diagnosis of HP by current consensus criteria thus limiting incorporation bias. The diagnostic criteria established by the American Thoracic Society was used in this study given that it incorporates the CHEST diagnostic criteria and emphasizes the importance of a biopsy in determining a diagnosis. Additionally, only patients who met criteria for definite or high probability HP were included which further strengthened the diagnosis of HP in these patients. There are limitations to this study that should be acknowledged. This was a retrospective study thus it is difficult to determine if antigen removal actually occurred, as the vast majority of patients in our study did not have data in the medical record to definitively determine whether the antigen was removed. Additionally, the compliance with concomitant administration of immunosuppression could not be assessed in this retrospective study. Finally, the cohort is from a single quaternary referral center with expertise in ILD thus inclusion of cohorts from other institutions with ILD expertise is required for external validation.

## Conclusion

Definitive diagnosis of HP with bronchoalveolar lavage and transbronchial biopsy may be beneficial when recommending antigen removal as transplant free survival is impacted only when this diagnosis is present. Survival is not affected with antigen identification in patients with a diagnosis other than HP thus removal my not be impactful in those cases.

### Electronic supplementary material

Below is the link to the electronic supplementary material.

Supplementary Material 1

## Data Availability

All data generated or analyzed during this study are included in this published article.
