# Supplementary material for: Impact of antigen identification on transplant free survival in interstitial lung disease
Source: BMC Pulm Med. 2023 Oct 26;23:404. doi: 10.1186/s12890-023-02724-w (PMC10601144; doi:10.1186/s12890-023-02724-w)
Supplement: Supplementary file 1 — Supplementary Material 1 [file 12890_2023_2724_MOESM1_ESM.docx]

Supplementary Appendix 1:

Exposure Questionnaire:

- Residence:
  - Type of Residence (House, Apartment, Mobile Home)
  - Age of Residence
  - Years in Current Residence
  - If applicable, type, age, and years in prior residences
  - Type of Foundation (concrete slab vs pier and beam)
    - If pear and beam, last crawl space inspection and findings
- Prior water damage to the home:
  - Location of water damage
  - Type of water damage
  - Duration of water damage
  - Type of remediation
- Visible mold in the home:
  - Duration of exposure
  - Location of exposure
  - Type of remediation
- Type of Air conditioner (Central, Window unit):
  - Any mold damage
- Pool use
- Hot Tub use
- Sauna use
- Humidifier use
- CPAP or BiPAP use
- Birds in the home
- Feather products in the home
- Hobbies
- Occupational History
- Wood working
- Metal working
- Gardening
  - If so, presence and use of compost heap
- Wind instrument use
